# Compound Sophorae Decoction Alleviates Ferroptosis in Colitis Rats via Activating Keap1/Nrf2/GPX4 Signaling Pathway

**DOI:** 10.1155/grp/6298090

**Published:** 2025-12-28

**Authors:** Jingbo Wang, Qianyun Chen, Si Chu, Feng Zhu, Lijuan Zhang, Zaifeng Yi, Jingjing Li, Desheng Hu, Heng Fan, Ting Yu

**Affiliations:** ^1^ Department of Integrated Traditional Chinese and Western Medicine, Union Hospital, Tongji Medical College, Huazhong University of Science and Technology, Wuhan, Hubei Province, China, hust.edu.cn; ^2^ Health Management Center, Hubei Provincial Hospital of Integrated Chinese and Western Medicine, Wuhan, Hubei Province, China; ^3^ Department of Gerontology, Hubei Provincial Hospital of Integrated Chinese and Western Medicine, Wuhan, Hubei Province, China

**Keywords:** compound sophorae decoction, ferroptosis, Keap1/Nrf2/GPX4 signaling pathway, ulcerative colitis

## Abstract

**Background:**

Compound sophorae decoction (CSD) has been extensively applied in clinic for the treatment of ulcerative colitis (UC). However, the effect and precise therapeutic mechanism have not been fully clarified. In this study, we systematically explored the protective efficacy and the underlying molecular mechanisms of CSD against UC.

**Methods:**

The main constituents of CSD were analyzed by HPLC‐MS/MS and TLC. H_2_O_2_‐treated Caco‐2 cells were employed to investigate the impact of CSD on ferroptosis and its underlying mechanism. The impacts of CSD on inflammation, oxidative stress, and ferroptosis *in vitro* were evaluated by ELISA, biochemical detection, or fluorescence probe. The UC model was established in rats by administering 5% DSS in the drinking water. The effects and safety of CSD on DSS‐induced colitis were evaluated through daily body weight, DAI, colon length, and HE staining. In addition, cytokines (IL‐1*β*, IL‐6, TNF‐*α*, and TGF‐*β*) and ferroptosis‐associated parameters (iNOS and PTGS2) were detected by ELISA. Antioxidant and oxidant enzyme activities (SOD, GSH, MDA, and NO) and lipid ROS in serum and colon tissue were measured by biochemical kit. Ferroptosis was determined by analysis of ferroptosis‐associated proteins (Keap1, Nrf2, GPX4, and SLC7A11).

**Results:**

Then, 10 main active components were identified in CSD. CSD significantly attenuated DSS‐induced intestinal injury and inflammation. Moreover, CSD notably decreased oxidative stress and lipid peroxidation. Mechanistically, CSD suppressed ferroptosis in DSS‐induced UC and upregulated GPX4 and SLC7A11 expression through the activation of Nrf2 signaling in DSS‐induced rats.

**Conclusions:**

Collectively, this study demonstrated that CSD ameliorates ferroptosis in DSS‐induced UC rats, with its protective effects attributed to the activation of the Keap1/Nrf2/GPX4 signaling pathway.

## 1. Introduction

Ulcerative colitis (UC), a form of inflammatory bowel disease (IBD), is characterized by continuous mucosal inflammation, resulting in colonic ulcerations and bloody diarrhea [1]. The pathogenesis is multifactorial, involving environmental factors, genetic susceptibility, epithelial barrier defects, intestinal flora alteration, aberrant immune responses, and dysregulated cell death pathways [2]. In recent years, the morbidity of UC has still been increasing, while the efficacy of conventional drugs is not satisfactory, which has become one of the primary threats to international public health problems [3, 4].

Ferroptosis is an iron‐dependent cell death and is involved in multiple pathological conditions. Different from apoptosis, ferroptosis is triggered by the accumulation of intracellular lipid peroxidation and redox imbalance. Glutathione (GSH) depletion, glutathione peroxidase 4 (GPX4) inactivation, and lipid reactive oxygen species (ROS) accumulation are central regulators of ferroptosis. Accumulating evidence has demonstrated that ferroptosis is implicated in the pathogenesis of diverse diseases, including IBD, cancer, cardiomyopathy, acute liver injury, acute kidney injury, and ischemia–reperfusion injury [5–7]. Xu et al. revealed that ferroptosis contributes to the pathological development of colitis in mice, thereby inducing the death of intestinal epithelial cells (IECs) [8].

Previous studies suggested that oxidative stress exhibits a profound association with UC. Nuclear factor erythroid 2–related factor 2 (Nrf2) acts as a key transcriptional regulator that modulates the expression of genes harboring antioxidant response elements (AREs), with GPX4 being a prominent downstream target. Besides, the Kelch‐like ECH‐associated protein 1 (Keap1) also plays a pivotal role in antioxidant defense [9–12]. In recent years, mounting evidence has demonstrated that the activation of the Nrf2‐Gpx4 signaling axis exerts a significant inhibitory effect on ferroptosis in experimental colitis models [13]. Furthermore, it has been reported that triggering the P62‐Keap1‐NRF2 pathway leads to the inhibition of ferroptosis [14].

Compound sophorae decoction (CSD) is an effective prescription for the treatment of UC in clinic, which has the function of clearing heat and detoxification, cooling blood, and drying dampness. CSD is mainly composed of six traditional Chinese herbs, namely, *Sophora flavescens* Ait, *Sanguisorba officinalis* L, Indigo Naturalis, *Bletilla striat*a (Thunb.) Reichb f., *Glycyrrhiza uralensis* Fisch, and *Panax notoginseng* (Burk) F. H. Chen. Our previous research demonstrated that CSD has excellent anti‐inflammatory, antioxidant, antitumor, and immunomodulatory effects [15–17]. Notably, CSD effectively attenuates inflammatory and oxidative stress responses in experimental colitis. However, the effects of CSD on ferroptosis and the underlying mechanism of ferroptosis in UC remain poorly understood. In this study, DSS‐induced UC rats were utilized to evaluate the effects and potential mechanism of CSD on UC. Then, the role of CSD on ferroptosis‐related indicators in UC rats and H_2_O_2_‐induced Caco‐2 cells was further analyzed. Additionally, the modulation of CSD on oxidative stress–associated biomarkers and pivotal targets in the ferroptosis signaling pathway was systematically and comprehensively explored. This investigation aided in unraveling its potential molecular mechanisms and further validated the feasibility for its therapeutic application.

## 2. Materials and Methods

### 2.1. Preparation of CSD

CSD consists of *Sophora flavescens* Ait*, Sanguisorba officinalis* L, *Indigo Naturalis, Bletilla striata* (Thunb.) Reichb f., *Glycyrrhiza uralensis* Fisch, and *Panax notoginseng* (Burk) F. H. Chen. The above six herbs were combined in the proportion of 15:15:10: 10:3:3, weighing 103 g in total. Except for *Panax notoginseng*, all herbs were first boiled with strong heat for 30 min; then, gentle heat was used for another 30 min. The decoction is collected after filtration, and the remaining drug residue was decocted with water again. Mix all the decoction, add *Panax notoginseng* powder, and stir well. Finally, all the decoction was continued to decoct and concentrated to the crude drug volume of 0.735 g/mL at 4°C for further use.

### 2.2. Preparation of CSD Containing Serum

The stored CSD at 4°C was rewarmed and mixed. Rats in the traditional Chinese medicine treatment group were given 2 mL of concentrated CSD by gavage every day for 7 days. On the 8th day, all rats were anesthetized by intraperitoneal injection of 3% pentobarbital sodium, followed by dissection under sterile conditions. Blood was collected from the carotid artery and centrifuged at 3000 rpm for 15 min at 4°C. Then, the serum in the upper layer was carefully collected. Ultimately, the serum underwent filtration, sterilization, and inactivation processes, whereafter it was aliquoted into microcentrifuge tubes and cryopreserved at −80°C for subsequent utilization. In addition, rats in the control group were administered 2 mL of PBS by gavage every day for 7 days. Then, the control serum was obtained by the above method for use.

### 2.3. HPLC‐MS/MS and TLC Analyses of CSD

The CSD was prepared and concentrated into 1.0 g/mL. The CSD was precipitated overnight at 4°C; then, the supernatant was collected on the following day. The resulting supernatant (200 *μ*L) was supplemented with 1 mL of methanol: water (8:2, V:V), vortexed thoroughly, and centrifuged at 20,000 *g* for 10 min at 4°C. The supernatant after centrifugation was filtered with a 0.22‐*μ*m filter membrane. The standard was diluted with pure methanol as the standard working solution. The final filtrate was taken for analysis.

### 2.4. Establishment of UC Rat Model and Treatment Strategy

Then, 24 SPF SD male rats were randomly assigned to four groups: the normal control group, model group, CSD group, and mesalazine group (*n* = 6). After 1 week of adaptive feeding, except for the normal control group, other groups of rats were administered 5% DSS (MP Biomedicals, No. 160110) for 1 week. After DSS successful modeling, rats in the CSD group were given 2 mL concentrated decoction of CSD by gavage daily for a week. Rats in the mesalazine group received mesalazine treatment (0.42 g/kg) by gavage once per day for a week. Meanwhile, the control group and the model group were gavaged with the same volume of PBS.

### 2.5. Construction of Caco‐2 Cell Model Induced by H_2_O_2_

Caco‐2 cells are a type of human clonal colon adenocarcinoma cells structurally and functionally similar to small IECs. The Caco‐2 cell line utilized in this study was procured from the ATCC cell bank. The cell line was cultivated in DMEM high glucose medium containing 10% fetal bovine serum (FBS) and 1% penicillin–streptomycin solution. Caco‐2 cells in a favorable growth state were trypsinized and then seeded into 6‐well plates at a density of 3 × 10^5^ cells per cm^2^.When the cells grew to 80%–90% confluence, the old cell culture medium was removed, the new culture medium and corresponding drugs were added according to different treatment conditions, the culture was continued for 12 h. After 12 h, the culture medium was aspirated, and the cells were washed with PBS; then, all cells except the control group were exposed to 350 *μ*M H_2_O_2_ to induce inflammation and oxidative stress.

### 2.6. Assessment of Disease Severity in UC Rats

From the beginning of DSS modeling, the diet, mental status, weight gain, and stool characteristics of rats in each group were continuously observed, and the disease activity index was evaluated until the end of drug administration.

### 2.7. ROS Fluorescent Probe Detection

DCFH‐DA probe medium and hydrogen donor were prepared in accordance with the kit′s instructions. Negative control wells, positive control wells, and sample wells were set; then, the corresponding probe and medium were added according to the instructions. Negative control wells were added with serum‐free medium only, positive control wells were added with serum‐free medium with probe and hydrogen donor, and sample wells were added with serum‐free medium and probe only. All cells were incubated at 37°C for 45 min. When green fluorescence was observed in the cells under the fluorescence microscope, the culture medium was aspirated, and the cells were rinsed with PBS. The collected cells were suspended in 1.5‐mL EP tubes, washed twice with PBS, and centrifuged at 1000 rpm for 5 min. After resuspension in PBS, the FITC fluorescence was detected by flow cytometry.

### 2.8. Hematoxylin and Eosin (H&E) Staining

Rats were anesthetized with sodium pentobarbital (90 mg/kg, intraperitoneal injection), sacrificed by cervical dislocation, and colon tissues were immediately excised. A portion of the colon tissue was taken, fixed in 4% paraformaldehyde overnight, then embedded in paraffin. The paraffin‐embedded colon tissues were cut into 4‐*μ*m thick sections. The sections were baked at 37°C overnight and subsequently at 75°C for 2 h. They were then dewaxed and dehydrated in xylene followed by a descending gradient of ethanol. For H&E staining, the sections were subjected to hematoxylin staining for 3 min followed by rinsing with tap water. Next, the nucleus and cytoplasm were stained with H&E, respectively. The sections were then dehydrated in the reverse order of dewaxing. After dehydration, the slides were mounted with neutral gum and observed the pathological changes of the colon under the microscope.

### 2.9. Western Blotting

Western blotting analysis of colon tissue was conducted following the previously described protocol. Anti‐Keap1 (1:1000, Abclonal, A1820), anti‐Nrf‐2 (1:1000, Abclonal, A21176), anti‐GPX4 (1:1000, Abclonal, A11243), anti‐SLC7A11 (1:1000, Abclonal, A25302), anti‐NF‐*κ*Bp65 (1:2000, Cell Signaling Technology, 8242), and anti‐pNF‐*κ*Bp65 (Ser536) (1:1000, Cell Signaling Technology, 3033) antibodies were used as primary antibodies, respectively. The protein expression was normalized to *β*‐actin.

### 2.10. Biochemical Detection

The contents of MDA (A003‐1‐2), GSH (A006‐2‐1), SOD (A001‐2‐2), NO (A013‐2‐1), and MPO (H508‐1‐2) in rat colon and cell lysates were assessed using commercial kits. All reagent detections followed the manufacturer’s (Nanjing Jiancheng Bioengineering Institute) protocol. Method for extracting total protein from colon tissue in biochemical testing: Accurately weigh the tissue weight. Add nine times the volume of normal saline according to the ratio of weight (g): volume (mL) = 1:9. Chop the tissue, prepare the homogenate in an ice water bath, centrifuge at 2500–4000 revolutions per minute for 10 min, and take the supernatant, which is the 10% homogenate supernatant, for testing.

### 2.11. ELISA Detection

The levels of IL‐1*β* (E‐EL‐R0012), IL‐6 (E‐EL‐R0015), TNF‐*α* (E‐EL‐R2856), IL‐10 (E‐EL‐R0016), TGF‐*β* (E‐UNEL‐R0054), iNOS (E‐EL‐R0520), and PTGS2 (E‐EL‐R0792) in serum and colon tissue of UC rats were assessed using ELISA kits following the manufacturer′s (Elabscience) protocols. Method for preparing colon tissue in ELISA testing is as follows: Rinse the colon tissue with precooled PBS, weigh it, and then cut it into pieces. Add nine times the volume of PBS to the homogenizer and thoroughly grind the cut tissue on ice. To further lyse the tissue cells, perform ultrasonic disruption on the homogenate. Finally, centrifuge the colon tissue homogenate at 4°C, 5000 × *g* for 5–10 min, and take the supernatant for detection. Method for preparing serum samples in ELISA testing is as follows: After blood was collected from the orbital cavity, the whole blood samples of the rats were left at room temperature for 1 h. Then, they were centrifuged at 1000 × *g* for 20 min at 4°C, and the supernatant was collected for detection.

### 2.12. Statistical Analysis

All data were analyzed by GraphPad Prism 8.0 software (GraphPad Software, Inc.) and presented as means ± SD. When all the data within each group were normally distributed and equal variances, we would use one‐way ANOVA (analysis of variance). If there were nonnormally distributed data or unequal variances among different groups, we would use the Kruskal–Wallis *H* test. For pairwise comparisons among multiple sets of data, we employ the Bonferroni post hoc test. *p* < 0.05 was considered statistically significant.

## 3. Results

### 3.1. CSD Alleviated DSS‐Induced Experimental Colitis

First, the active ingredients of CSD were identified by HPLC‐MS/MS and TLC. Then, 10 active ingredients, including matrine, oxymatrine, gallic acid, liquiritin, glycyrrhizic acid, ginsenoside Rb1, notoginsenoside R1, indigo, indirubin, and Bletilla striata (Thunb) Reichb.f., were identified, which can exert extensive biological effects (Supporting Information 1: Figure S1). To assess the biological effects and therapeutic potential of CSD, a DSS‐induced UC rat colitis model was established (Figure 1a). After 7 days of modeling with 5% DSS, the rats showed obvious mucoid bloody stool (Figure 1b), weight loss (Figure 1c), and significantly increased DAI score [15, 18] (Figure 1d). However, the clinical symptoms and DAI scores were effectively reversed after CSD treatment compared with the model group (Figure 1c,d). Moreover, the colon length of the DSS group was markedly shorter than that of the control group. After treatment with CSD, the shortened colon of UC rats was effectively alleviated (Figure 1e,f). In addition, H&E staining revealed that the colon tissue in the DSS group was obviously damaged, with massive inflammatory cell infiltration, colonic epithelial goblet cell depletion, intestinal crypt structural disruption, gland disorder, and ulcer formation. However, CSD significantly improved the pathological changes of the intestinal mucosa (Figure 1g). Myeloperoxidase (MPO) has been established as a key local mediator of inflammatory responses in UC [19]. We found that the activity of MPO was significantly higher in the colon tissue and serum in the DSS group, which was notably reduced after CSD treatment (Figure 1h). Overall, CSD had a significant therapeutic effect on DSS‐induced colitis.

Figure 1CSD alleviated DSS‐induced colitis. (a) The schematic of the UC model and drug treatment. (b) The UC model of rats was successfully constructed. (c) The weight change curve of four groups of rats over time. (d) Changes in DAI scores in four groups of rats over time. (e, f) Changes in colon morphology (e) and length (f). (g) Representative HE staining of colon tissues in four groups of rats. Scale, 100 *μ*m. (h) MPO activity in colon tissue and serum of rats. ^##^ *p* < 0.01 vs. the control group.  ^∗^ *p* < 0.05 and  ^∗∗^ *p* < 0.01 vs. the model group (*n* = 6). Data are from three independent experiments.

**Figure:**
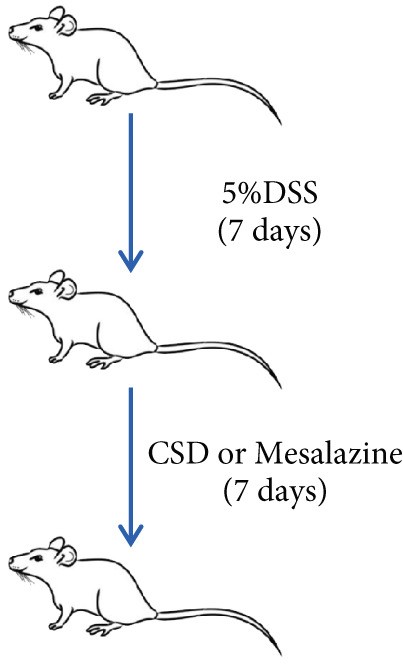
(a)

**Figure:**
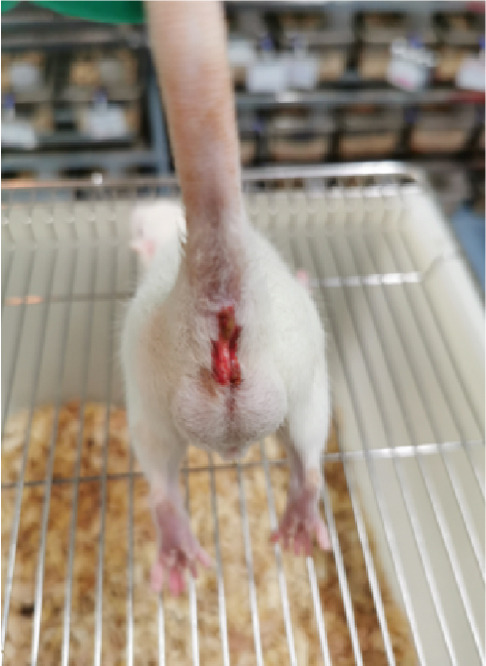
(b)

**Figure:**
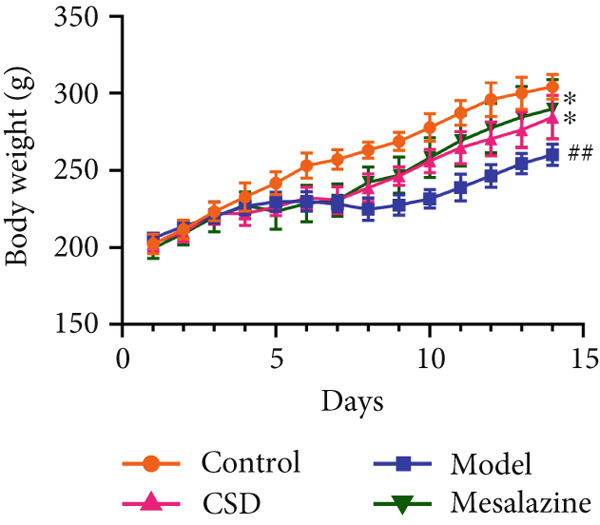
(c)

**Figure:**
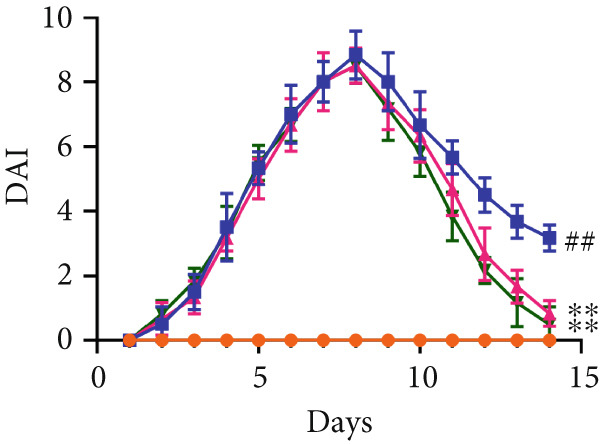
(d)

**Figure:**
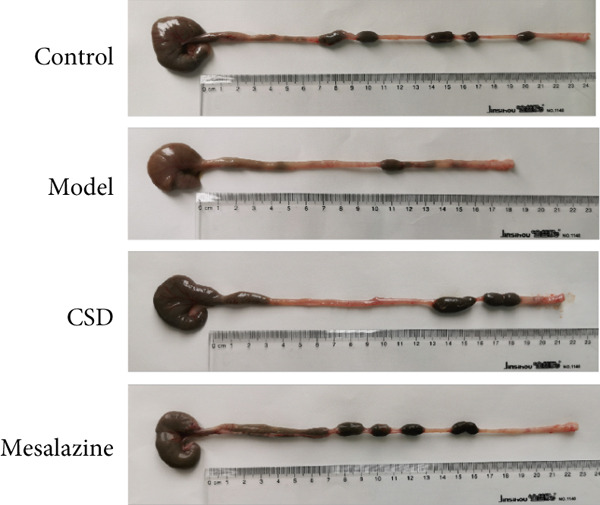
(e)

**Figure:**
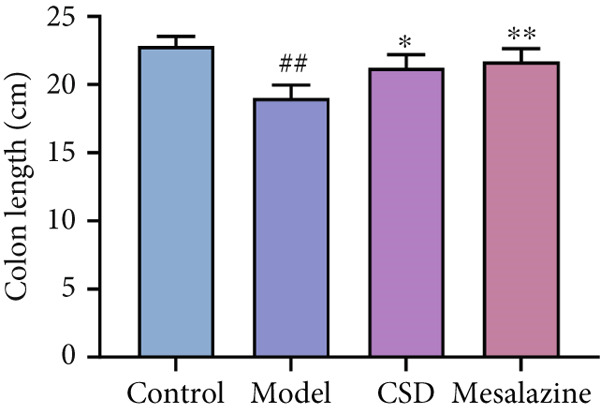
(f)

**Figure:**
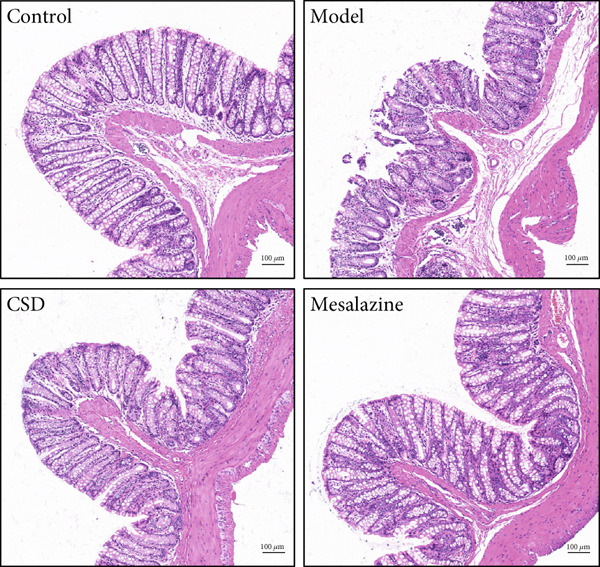
(g)

**Figure:**
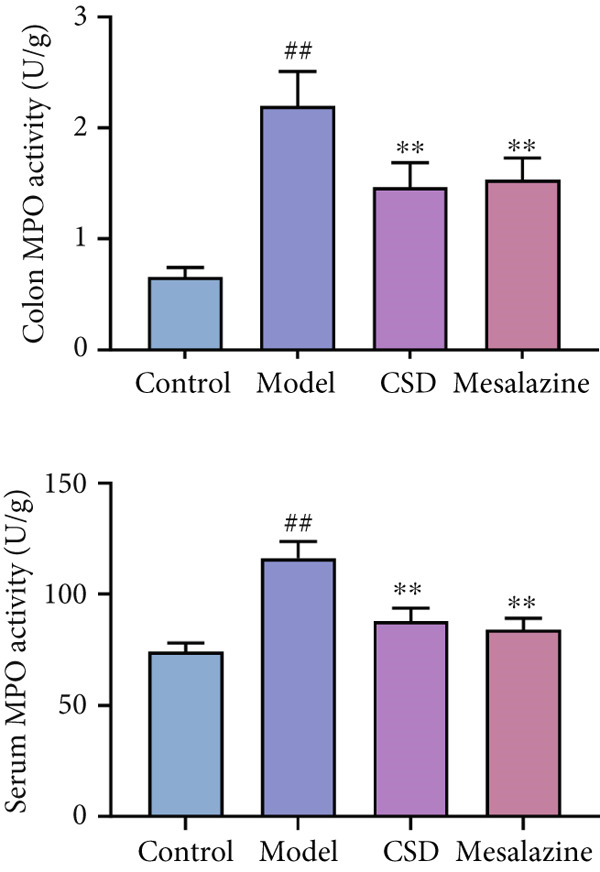
(h)

### 3.2. CSD Attenuated Inflammation and Oxidative Stress Response in UC

To investigate the effect of CSD on inflammatory status, the levels of cytokines in the colon tissues and serum were detected. Compared to the control group, the model group exhibited upregulated levels of proinflammatory cytokines including IL‐1*β*, IL‐6, and TNF‐*α*, and a reduced level of anti‐inflammatory cytokines IL‐10 and TGF‐*β*, suggesting an enhanced inflammatory response (Figure 2a,b). CSD administration obviously reversed the expression patterns of abovementioned cytokines, suggesting an inhibitory effect of CSD on inflammation in the UC rats (Figure 2a,b). NF‐*κ*B p65 is also a key inflammatory mediator. The WB results showed that compared with the DSS group, the levels of NF‐*κ*B p65 and its phosphorylation p‐p65 in colon tissues of CSD groups were downregulated (Figure 2c). Excessive oxidative stress is not only involved in the pathogenesis of UC but also a vital process in developing ferroptosis. Consequently, we also investigated oxidative stress‐related factors in colon tissue and serum of rats. The results demonstrated that the DSS group exhibited significantly diminished levels of GSH and superoxide dismutase (SOD), concomitant with markedly elevated concentrations of malondialdehyde (MDA) and nitric oxide (NO). Fortunately, these trends were markedly reversed by CSD treatment (Figure 2d,e). In addition, these results were further verified in the cultured Caco‐2 cells in vitro. Similar results were obtained for the expression of cytokines (Supporting Information 2: Figure S2a) and the activities of GSH, MDA, NO, and SOD (Supporting Information 2: Figure S2b). In conclusion, CSD inhibited inflammatory response and oxidative damage in UC.

Figure 2CSD inhibited inflammatory response and oxidative damage in DSS‐induced colitis. (a, b) ELISA was used to detect the expression levels of IL‐1*β*, IL‐6, TNF‐*α*, IL‐10, and TGF‐*β* in colon tissue (a) and serum (b). (c) The expression of NF‐*κ*Bp65 and p‐NF‐*κ*Bp65 in colon tissues was detected by WB. (d, e) The levels of GSH, SOD, MDA, and NO in colon tissue (d) and serum (e) of rats. ^##^ *p* < 0.01 vs. the control group.  ^∗^ *p* < 0.05 and  ^∗∗^ *p* < 0.01 vs. the model group (*n* = 6). Data are from three independent experiments.

**Figure:**
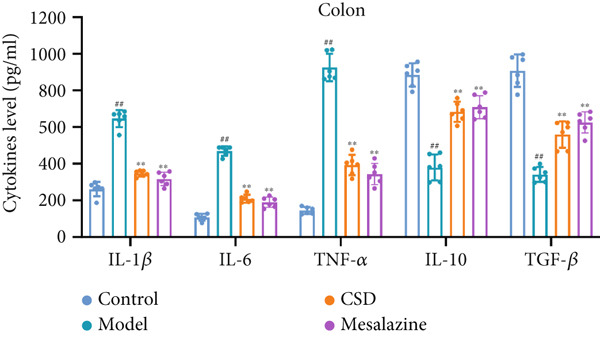
(a)

**Figure:**
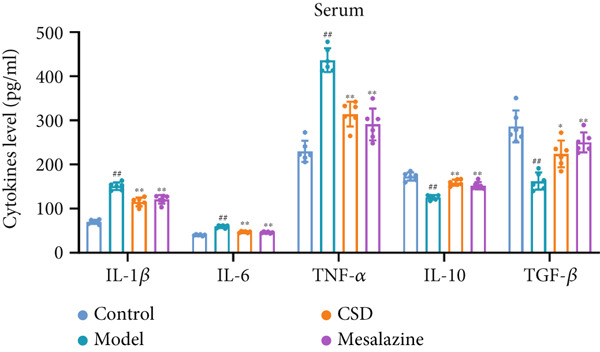
(b)

**Figure:**
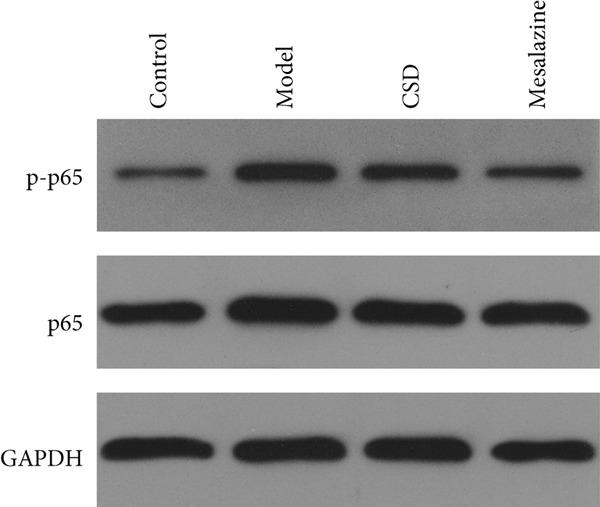
(c)

**Figure:**
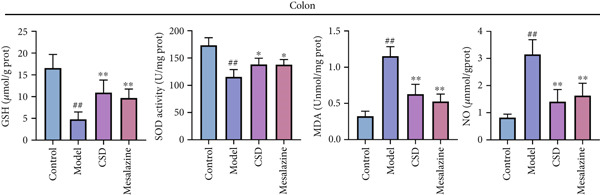
(d)

**Figure:**
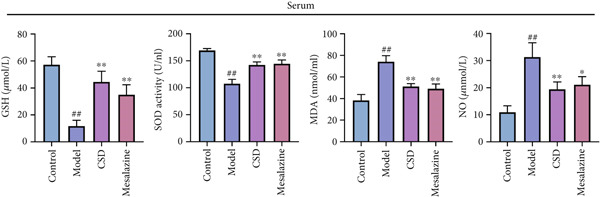
(e)

### 3.3. CSD Inhibited Ferroptosis by Regulating Keap1/Nrf2/GPX4 Pathway in UC

It is well known that ferroptosis plays an important role in the pathogenesis of UC. The accumulation of ROS, lipid peroxidation, GSH depletion, and upregulation of PTGS2 expression are key factors in ferroptosis. The inhibitory effect of CSD on ferroptosis was further verified in vitro. The experimental results showed a significant increase in ROS levels in the model group. However, the production of ROS was markedly reversed by serum containing CSD in a dose‐dependent manner (Figures 3a, 3b, and 3c). As shown in Figure 3d, the expression of PTGS2 exhibited a consistent trend as well. These results demonstrated that serum containing CSD effectively suppressed ferroptosis in Caco‐2 cells.

Figure 3CSD drug‐containing serum inhibited ferroptosis in H_2_O_2_‐treated Caco‐2 cells. (a) Flow cytometry was used to detect the production of ROS labeled by the fluorescent probe DCFH‐DA in Caco‐2 cells. (b) Statistical analysis of ROS percentage content in Caco‐2 cells. (c) Statistical analysis of the mean fluorescence intensity of FITC in Caco‐2 cells. (d) The expression level of PTGS2 in Caco‐2 cells was detected by ELISA. ^##^ *p* < 0.01 vs. the control group.  ^∗^ *p* < 0.05 and  ^∗∗^ *p* < 0.01 vs. the model group (*n* = 4–5). Data are from three independent experiments.

**Figure:**
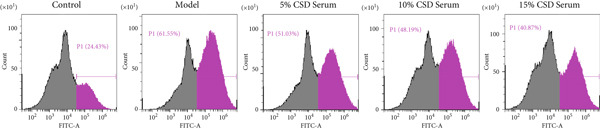
(a)

**Figure:**
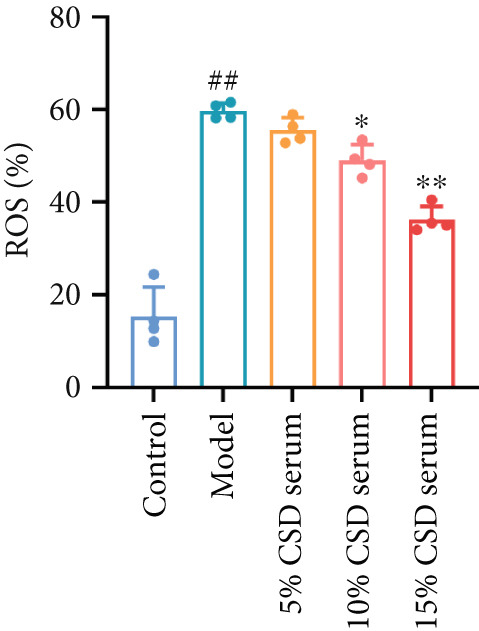
(b)

**Figure:**
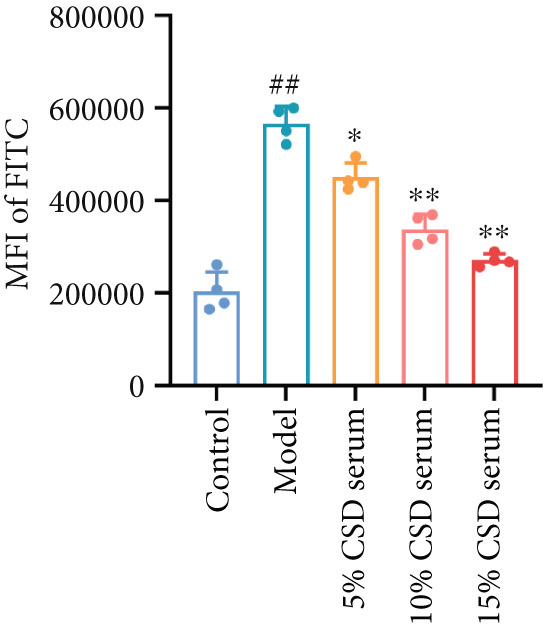
(c)

**Figure:**
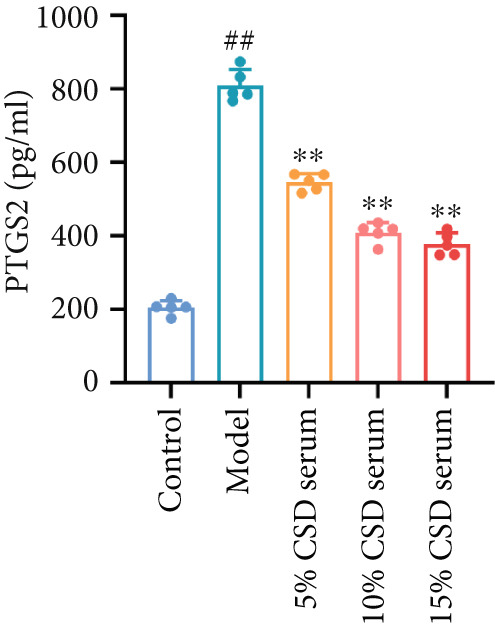
(d)

In order to further explore the mechanism of CSD inhibiting ferroptosis, the key markers were examined in vivo, including GPX4, SLC7A11, PTGS2, and iNOS. As shown in Figure 4a,b, the expressions of PTGS2 and iNOS were significantly upregulated after administration with DSS, whereas CSD markedly alleviated this change. In contrast, CSD significantly enhanced the levels of GPX4 and SLC7A11 in DSS rats (Figure 4c). GPX4, a vital regulatory enzyme of ferroptosis, is also a downstream molecule of Nrf2. Besides, Nrf2 is inversely regulated by the upstream gene Keap1. Research has confirmed that the Keap1/Nrf2 pathway was involved in the regulation of ferroptosis. As shown in Figure 4c, DSS treatment led to a decline in the expression of Nrf2 in the colon tissues of rats, while the expression of Keap1 was increased. However, the above indexes were significantly reversed after CSD treatment (Figure 4c). Subsequently, the biosafety of CSD was further analyzed. H&E staining suggested that there were no significant histological differences in the lung, heart, liver, spleen, and kidney between the CSD treatment and control groups (Supporting Information 3: Figure S3). Collectively, these findings demonstrated that ferroptosis participates in the pathogenesis of UC; moreover, CSD inhibits ferroptosis through the Keap1/Nrf2/GPX4 pathway.

Figure 4CSD alleviated ferroptosis in DSS‐induced colitis dependent on Keap1‐Nrf2 signaling. (a, b) The expression levels of PTGS2 (a) and iNOS (b) in colonic tissue (left) and serum (right) were detected by ELISA (*n* = 6). (c) The expression levels of Keap1, Nrf2, GPX4, and SLC7A11 in colon tissues of rats were detected by WB (*n* = 3). ^##^ *p* < 0.01 vs. the control group.  ^∗∗^ *p* < 0.01 vs. the model group. Data are from three independent experiments.

**Figure:**
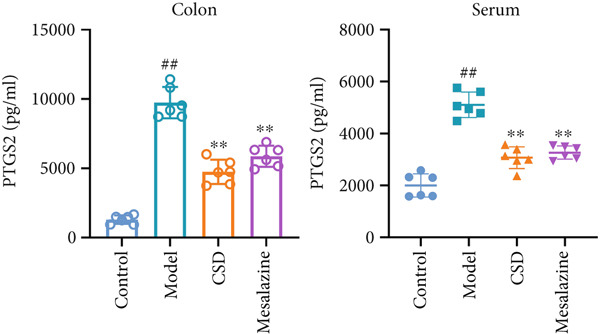
(a)

**Figure:**
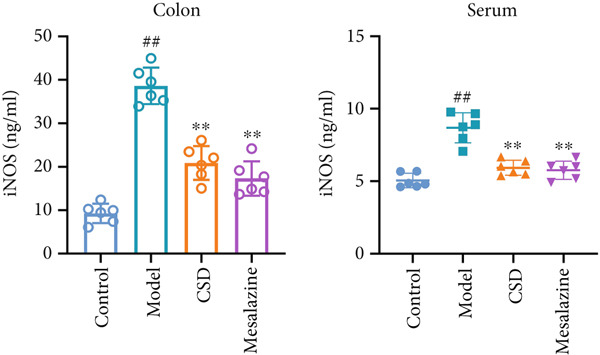
(b)

**Figure:**
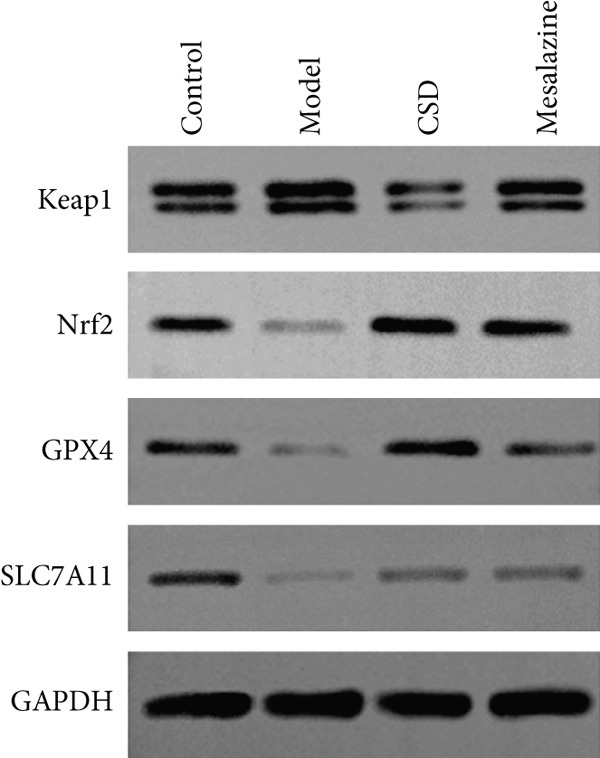
(c)

## 4. Discussion

UC is a nonspecific intestinal disease characterized by chronic inflammation of the colonic mucosa. The clinical manifestations include abdominal pain, diarrhea, mucous and bloody stool, and tenesmus [20, 21]. Currently, the clinical drugs for UC mainly include mesalazine, glucocorticoids, immunosuppressants, and TNF‐*α* monoclonal antibodies. However, these drugs have limited efficacy and many side effects [22, 23]. Therefore, there is still a large and arduous scientific research demand for the development of new drugs.

Recently, the treatment of UC with traditional Chinese medicine has received increasingly widespread attention [24, 25]. CSD is an effective herbal decoction for the treatment of UC. In this study, HPLC‐MS/MS and TLC were used to identify the main active components of CSD, including matrine, oxymatrine, notoginsenoside R1, ginsenoside Rb1, gallic acid, indigo, indirubin, glycyrrhizic acid, liquiritin, and *Bletilla striata*. Several studies have shown that matrine, oxymatrine, indigo, gallic acid, and other monomers have been used in the treatment of UC, and can attenuate experimental colitis through anti‐inflammatory and immune regulation [26–34]. Previous studies have demonstrated that CSD has good anti‐inflammatory and antioxidant effects and can effectively relieve the clinical symptoms of UC [15–17]. Similar results were obtained in the current study. This study revealed the protective effect of CSD on UC, which improved body weight, increased colon length, ameliorated colon pathological changes, and DAI scores. MPO is a marker associated with neutrophils. In colon samples, the level of MPO was significantly elevated in the DSS group compared to the healthy control group [19]. Similarly, in our study, CSD effectively suppressed the expression of MPO and inflammatory cytokines in UC rats. These findings suggested that CSD could protect against DSS‐induced colitis by relieving inflammation and intestinal epithelial injury.

The pivotal molecular mechanism underlying ferroptosis resides in the dysregulation between lipid peroxidation damage and antioxidant defense of the cell membrane. Our study also confirmed that the level of oxidative stress enhanced after DSS challenge. In contrast, CSD significantly reduced MDA and NO levels while elevating GSH and MDA contents. The results showed that CSD has a prominent antioxidant defense capacity. Stimulation of H_2_O_2_ can induce abnormal ROS elevation and lipid peroxidation. Our research suggested that serum containing CSD addition markedly decreased the expression of ROS in H_2_O_2_‐induced Caco‐2 cells in a dose‐dependent manner. Furthermore, we found that CSD administration obviously restored the expression of ferroptosis‐associated markers (PTGS2 and iNOS) in the colon to homeostatic levels after DSS challenge. Therefore, we proposed that CSD may inhibit ferroptosis by modulating the levels of cellular lipid peroxidation.

The SLC7A11 and GPX4 are the main target proteins involved in ferroptosis. SLC7A11 is a transporter protein in System Xc‐, which plays a key role in GSH synthesis [35–37]. GPX4 protects against lipid peroxidation and cell ferroptosis. Mayr et al. demonstrated that IECs in Crohn′s disease (CD) manifest compromised GPX4 enzymatic activity and hallmarks of lipid peroxidation [38]. Moreover, curculigoside, an active phytochemical isolated from *Curculigo orchioides* Gaertn, displayed protective efficacy against ferroptosis in UC through the upregulation of GPX4 expression [39]. In our study, strong lipid peroxidation was observed in the colon tissue of DSS‐induced UC rats, accompanied by a decrease in GPX4 and SLC7A11 levels. However, we found a significant increase in GPX4 and SLC7A11 levels after CSD treatment, indicating that ferroptosis was inhibited. These findings illustrated that the inhibition of ferroptosis by CSD is associated with the upregulation of GPX4 and SLC7A11 expression, which encourages us to further explore the ferroptosis mechanism by which CSD alleviates UC.

Studies have shown that Nrf2 and GPX4 are closely related. As a pivotal transcription factor governing the cellular antioxidant response, Nrf2 has been demonstrated to exert a central role in regulating ferroptosis, which can induce the production of the antioxidant enzyme GPX4 [13, 40, 41]. At the same time, Nrf2 participates in modulating the expression of ferroptosis‐associated genes, such as SLC7A11 and GPX4 [42]. Many studies have pointed out that ferroptosis could be mitigated by activating the Nrf2 signaling pathway, thereby ameliorating IBD [13, 43–45]. It has been reported that grain worm extract can effectively protect experimental colitis by regulating Nrf2‐dependent antioxidant responses [46]. Previous studies indicated that the inhibitory impacts of APS on IECs ferroptosis were linked to the activation of the NRF2/HO‐1 signaling axis [43]. From our results, it was observed that the expression of Nrf2 in the UC rats was signally decreased, suggesting that the occurrence of ferroptosis in the colon may be caused by the inhibition of Nrf2. In contrast, after CSD addition, the level of Nrf2 was upregulated, alleviating ferroptosis in the colon.

In general, Nrf2 is mainly located in the cytoplasm and binds to Keap1 in a certain ratio to form a stable heterodimer [47]. Furthermore, Keap1 is the major inhibitory protein of Nrf2. As an E3 ligase adaptor element, Keap1 promotes the polyubiquitination of the Nrf2 protein, leading to proteasome‐dependent Nrf2 degradation [48]. Therefore, inhibition of Keap1 activity can inhibit the degradation of Nrf2 [49]. In this study, contrary to the trend of Nrf2, the expression of Keap1 was markedly elevated in the DSS group. Excitingly, CSD has been found to inhibit the expression of Keap1. Taken together, these data suggest that CSD could alleviate ferroptosis in UC by activating the Keap1/Nrf2/GPX4 signaling pathway. The activation of the Nrf2 signaling axis emerges as a promising therapeutic strategy for the prevention of UC.

Overall, our study confirmed that CSD prominently alleviated DSS‐induced UC, evidenced by reducing colon pathological damage, relieving inflammation, and oxidative stress. Moreover, CSD inhibited ferroptosis in UC by activating the Nrf2 signaling pathway. This study provides innovative insights and experimental evidence for the management of UC with traditional Chinese medicine.

**Nomenclature**
CSD: compound sophorae decoction
UC: ulcerative colitis
IBD: inflammatory bowel disease
CD: Crohn′s disease
DSS: dextran sulfate sodium salt
HE: hematoxylin–eosin
DAI: disease activity index
GSH: glutathione
SOD: superoxide dismutase
MDA: malondialdehyde
NO: nitric oxide
ROS: reactive oxygen species
MPO: myeloperoxidase
IECs: intestinal epithelial cells
GPX4: glutathione peroxidase 4
SLC7A11: solute carrier family 7 member 11
Keap1: Kelch‐like ECH‐associated protein 1
Nrf2: nuclear factor erythroid 2–related factor 2
IL‐1*β*
: interleukin‐1*β*
IL‐6: interleukin‐6
IL‐10: interleukin‐10
TNF‐*α*
: tumor necrosis factor‐*α*
TGF‐*β*
: transforming growth factor *β*
NF‐*κ*B p65: p65‐nuclear factor kappa B
iNOS: inducible nitric oxide synthase
PTGS2: prostaglandin–endoperoxide synthase 2

## Ethics Statement

All the experiments were performed according to the guidelines and study protocols of the Institutional Animal Care and Use Committee at Tongji Medical College, Huazhong University of Science and Technology (IACUC Number, S2341).

## Consent

The authors have nothing to report.

## Disclosure

All authors have read and approved the final manuscript for publication.

## Conflicts of Interest

The authors declare no conflicts of interest.

## Author Contributions

T.Y. and H.F. conceived and designed the research project. J.W., S.C., F.Z., L.Z., J.L., and Z.Y. carried out the study. J.W., T.Y., Q.C., and S.C. performed the statistical analysis. T.Y., J.W., and Q.C. drafted the manuscript. D.H. and T.Y. revised the manuscript. J.W., Q.C., and S.C. have contributed equally to this work.

## Funding

This study was supported by the National Natural Science Foundation of China, 10.13039/501100001809, 82305199 and 81774093.

## Supporting Information

Additional supporting information can be found online in the Supporting Information section.

## Supporting information


**Supporting Information 1** Figure S1: HPLC‐MS/MS and TLC of main ingredients in CSD. (a) Matrine. (b) Oxymatrine. (c) Gallic acid. (d) Liquiritin. (e) Glycyrrhizic acid. (f) Ginsenoside Rb1. (g) Notoginsenoside R1. (h) Indigo. (i) Indirubin. The left panel is the standard sample; the right panel is the CSD sample. (j) TLC of Bletilla striata (Thunb) Reichb.f., Lane 1 was a positive sample, the Lanes 2, 3, and 4 were aqueous extracts of CSD, and Lane 5 was a negative sample.

**Supporting Information 2** Figure S2: CSD drug‐containing serum reduced the levels of inflammatory cytokines and oxidative stress in H_2_O_2_‐treated Caco‐2 cells. (a) ELISA was used to detect the expression levels of IL‐1*β*, IL‐6, TNF‐*α*, IL‐10, and TGF‐*β*. (b) The levels of GSH, SOD, MDA, and NO in Caco‐2 cells were detected by biochemical kits. ^##^ *p* < 0.01 vs. the control group.  ^∗^ *p* < 0.05 and  ^∗∗^ *p* < 0.01 vs. the model group (*n* = 5). Data are from three independent experiments.

**Supporting Information 3** Figure S3: Evaluation of drug safety of CSD. HE staining was used to detect the histopathological changes of the lung, heart, liver, spleen, and kidney of UC rats in the control group and CSD treatment group. Scale, 100 *μ*m (*n* = 3).

## Data Availability

The data in this study is available from the corresponding author upon reasonable request.
