# Supplementary figures and images for: Compound Sophorae Decoction Alleviates Ferroptosis in Colitis Rats via Activating Keap1/Nrf2/GPX4 Signaling Pathway
Source: Gastroenterol Res Pract. 2025 Dec 28;2025:6298090. doi: 10.1155/grp/6298090 (PMC12752875; doi:10.1155/grp/6298090)

## Slide 1
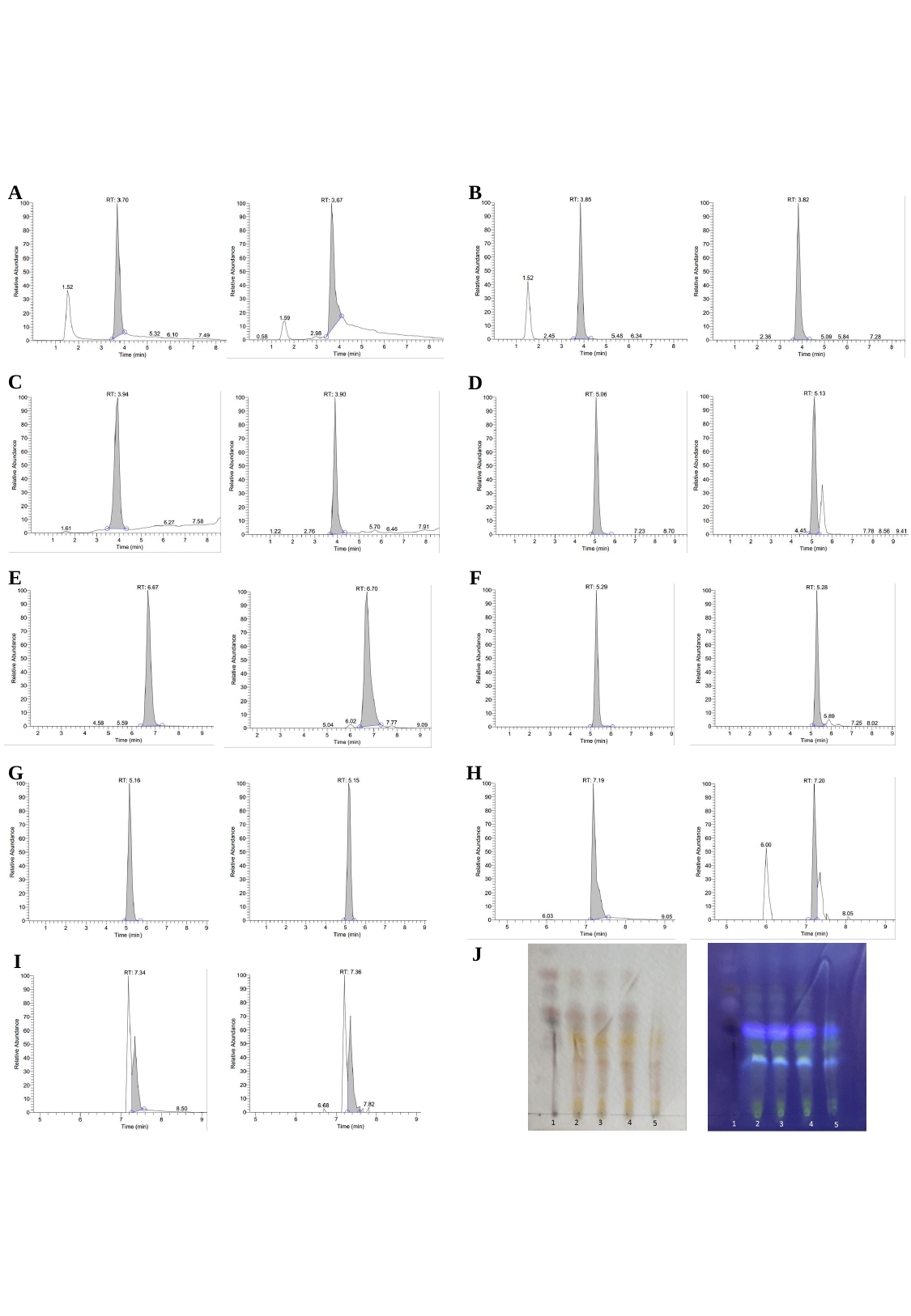

A
B
C
D
F
E
H
G
J
I

Supplement: Supplementary file 1 — Supporting Information 1 Figure S1: HPLC‐MS/MS and TLC of main ingredients in CSD. (a) Matrine. (b) Oxymatrine. (c) Gallic acid. (d) Liquiritin. (e) Glycyrrhizic acid. (f) Ginsenoside Rb1. (g) Notoginsenoside R1. (h) Indigo. (i) Indirubin. The left panel is the standard sample; the right panel is the CSD sample. (j) TLC of Bletilla striata (Thunb) Reichb.f., Lane 1 was a positive sample, the Lanes 2, 3, and 4 were aqueous extracts of CSD, and Lane 5 was a negative sample. [file GRP-2025-6298090-s001.pptx]

## Slide 1
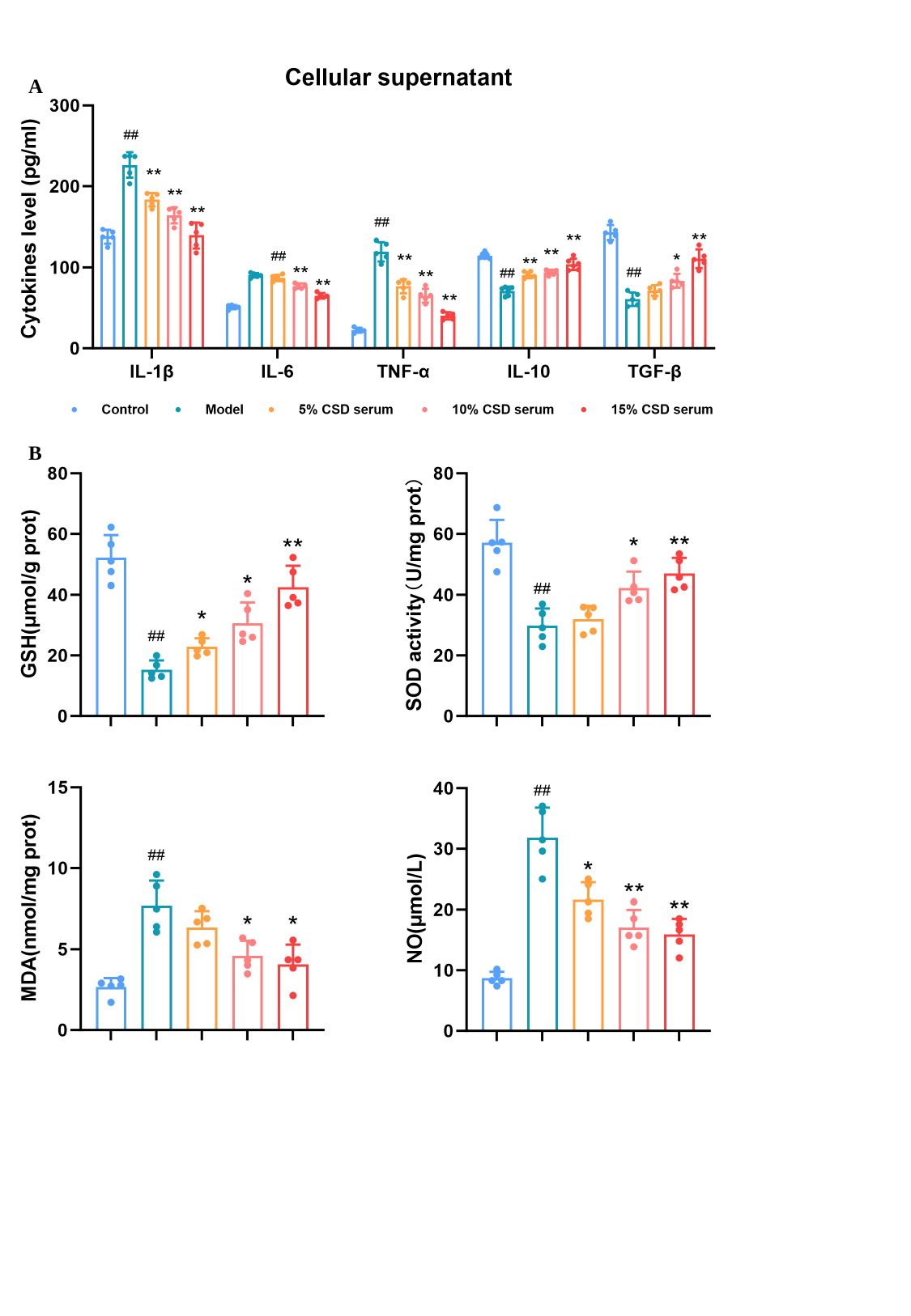

A
B

Supplement: Supplementary file 2 — Supporting Information 2 Figure S2: CSD drug‐containing serum reduced the levels of inflammatory cytokines and oxidative stress in H2O2‐treated Caco‐2 cells. (a) ELISA was used to detect the expression levels of IL‐1β, IL‐6, TNF‐α, IL‐10, and TGF‐β. (b) The levels of GSH, SOD, MDA, and NO in Caco‐2 cells were detected by biochemical kits. ## p < 0.01 vs. the control group. ∗ p < 0.05 and ∗∗ p < 0.01 vs. the model group (n = 5). Data are from three independent experiments. [file GRP-2025-6298090-s002.pptx]

## Slide 1
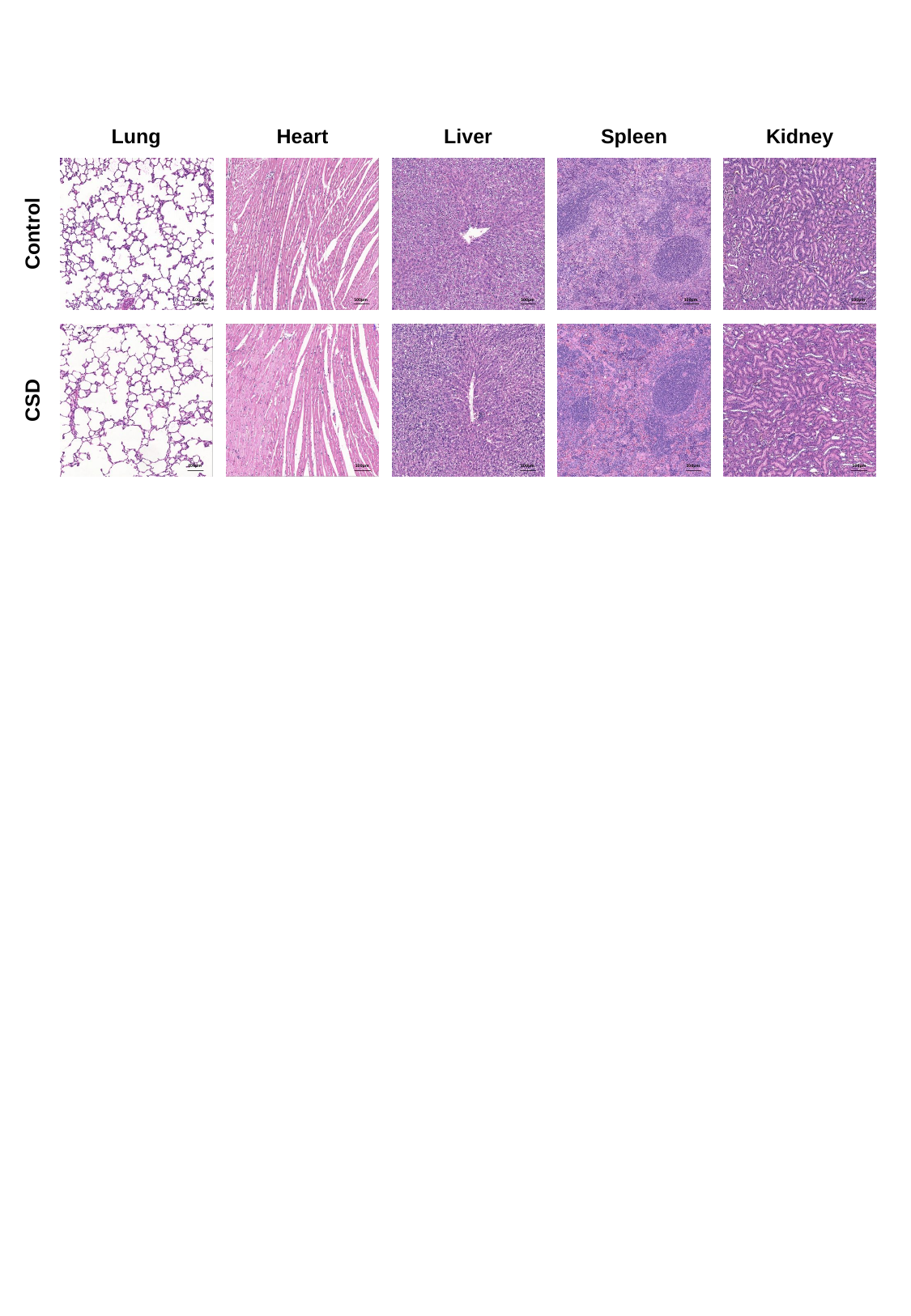

Liver
Spleen
Kidney
Lung
Heart
Control
100μm
100μm
100μm
100μm
100μm
CSD
100μm
100μm
100μm
100μm
100μm

Supplement: Supplementary file 3 — Supporting Information 3 Figure S3: Evaluation of drug safety of CSD. HE staining was used to detect the histopathological changes of the lung, heart, liver, spleen, and kidney of UC rats in the control group and CSD treatment group. Scale, 100 μm (n = 3). [file GRP-2025-6298090-s003.pptx]
